# IPPRAS Cryobank for the Conservation of Orthodox Seeds of Rare, Endangered, Medicinal, and Ornamental Plant Species—Current Research

**DOI:** 10.3390/plants13101354

**Published:** 2024-05-14

**Authors:** Olga Sergeevna Nits, Mariya Vladimirovna Sementsova, Ekaterina Sergeevna Osipova, Dmitry Viktorovich Tereshonok, Evgeny Aleksandrovich Gladkov

**Affiliations:** K.A. Timiryazev Institute of Plant Physiology, Russian Academy of Sciences (IPP RAS), Botanicheskaya 35, 127276 Moscow, Russia; nitsolga23071998@gmail.com (O.S.N.); guara2004@yandex.ru (M.V.S.); diman_ter_vi@mail.ru (D.V.T.); gladkovu@mail.ru (E.A.G.)

**Keywords:** cryobank, cryopreservation, germination, orthodox seeds, in vitro cultivation, rare, endangered, medicinal, ornamental plants, *Allium* spp., *Stipa* spp., *Veratrum* spp., *Cypripedium calceolus*

## Abstract

Plant cryobanks play a significant role in modern science and breeding. They contribute to the recovery of lost species, the emergence of new plant varieties, and help preserve and explore the diversity of the plant world. The IPPRAS Cryobank collection is constantly supplemented with new samples, while, at the same time, the stored samples are being monitored. In order to test seed germination, seeds of *Allium* and *Veratrum* species were thawed. Rare *Allium* species seeds, such as *A. nutans*, *A. schoenoprasum*, and *A. victorialis* were stored in liquid nitrogen for 17, 19, and 30 years, respectively. Long-term cryopreservation decreased germination rates for *A. nutans* from 96.55 to 50.00%, for *A. schoenoprasum* from 72.00 to 62.75%, and for *A. victorialis* from 90.00 to 83.05%. Seeds of a rare medicinal species, *Veratrum lobelianum*, were stored in liquid nitrogen for 18 years; the seed germination rate during this storage period has been significantly decreased from 75.00 to 14.81%. *V. nigrum* seeds were also collected and frozen in liquid nitrogen for 3 days. Short-term cryopreservation did not result in a statistically significant change in germination rates (from 79.71 to 82.69%). The seeds of an endangered ornamental species, *Cypripedium calceolus*, were collected and kept frozen for 3 days. After cryopreservation, the seeds were planted on three different media, as follows: ½ MS, MS with 10% coconut milk, and BM1. On ½ MS medium, 24.98% seeds formed protocorms, while on MS medium with 10% coconut milk, this number was 10.02%, and on BM1 medium, it was 15.02%, respectively; however, after 2.5 months, all of the protocorms died. Thus, it appears that the existing protocol for seed cryopreservation of *C. calceolus* needs further improvement. The size, weight, and free water content (WC) of six previously cryopreserved *Stipa* species and three *Allium* species were measured. For all the *Allium* and *Stipa* species studied, we found no correlation between seed size, WC, and cryotolerance. We also found no correlation between the life form, which reflects the water requirement of the species, and cryotolerance.

## 1. Introduction

A plant cryobank is an innovative institutional structure for the long-term storage of in vitro-derived germplasm, parts of plants, and seeds under liquid nitrogen (−196 °C) or its vapors (−135–180 °C). In order to perform its functions, a cryobank must provide special storage conditions that are created using the latest scientific developments and equipment [1,2,3]. The process of plant cryobank creation includes the collection of plant samples, their identification, passporting, processing, and freezing. Before freezing, special methods and substances (cryoprotectants) may be used to prevent damage to the cells from ice crystals and to preserve the genetic pool of the plants [4]. Frozen samples are stored in special containers to provide maximum protection from adverse environmental conditions. There are several types of plant cryobanks, depending on their scope and specialization. Some cryobanks are focused on conserving biodiversity, others on the preservation of rare and endangered species; there are also cryobanks that specialized in conserving food and economically important species. Cryobanks may be used to store seeds, pollen, and somatic and zygotic embryos, as well as plant parts such as roots, bulbs, tubers, buds, apical meristems, and suspension and callus cultures, among others [5].

Seeds are ideal reproductive structures for maintaining genetic variation in species, thus ensuring the conservation of the genetic pool. Seeds are also ideal for storage since they are small in volume, require little maintenance, and remain viable for long periods of time [6]. Studies of metabolism in frozen cultures of tissues, plant cells, and microorganisms indicate that in the not-deep freezing regime (from 0 to −25 °C), metabolic processes continue and lipid peroxidation is activated; as the temperature decreases below −25 °C, lipid hydrolysis processes intensify even further [7]. Even at −60 °C, dynamic processes take place in plant cell membranes [8]. At up to −130 °C, crystallization and recrystallisation processes are possible, which makes the long-term storage of frozen material impossible [9]. At lower temperatures, cell metabolism decreases to a level that may stop the degradation of tissues, including the possibility of restoring their biological functions after thawing [10]. Cryopreservation is particularly important for the conservation of short-lived orthodox seeds (microbiotics), which rapidly lose viability in conventional germplasm banks [11,12]. Research into the possibility of cryopreserving the seeds of endangered species collected in the wild began relatively recently, in the early 1980s, but it is already clear that this is a promising way to conserve the plant world’s genetic resources [13,14]. Studies have shown that the success of seed cryopreservation depends on the optimal moisture content, which is species-specific, as well as on a combination of the physical and chemical properties of the particular seeds [13,15,16].

The largest seed plant cryobanks are usually located in specialized botanical gardens, universities, and research centers; for instance, Plant and Seed Conservation Center, Iowa, USA; Arizona Botanical Garden, Arizona, USA; Kew Royal Botanic Gardens, London, UK; Millenium Seed Bank Project, West Sussex, UK; Global Crop Diversity Trust Facility in Wageningen, Netherlands; Vavilov Russian Institute of Plant Genetic Resources, Sankt-Petersburg, Russia; Norwegian Genetic Resource Centre in As, Norway. IPPRAS Cryobank does not have a large collection; however, it is one of the oldest cryobanks in Russia [17]. It was founded about 50 years ago by Dr. A.S. Popov, on the initiative of Prof. R.G. Butenko. In 1977, the first samples of the IPPRAS cryobank were placed in storage, in liquid nitrogen, for long-term cryopreservation [18]. The IPPRAS Cryobank is unique in the way that it functions as an independent research unit. While it carries out its own research program, IPPRAS Cryobank is also developing academic collaborations with a number of botanical gardens, agricultural research institutions, and universities [19,20]. A detailed history of IPPRAS Cryobank and its achievements is given by Yurieva et al. [19]. IPPRAS Cryobank contains collections of cell cultures, in vitro cultivated apical meristems, and the seeds of 457 species of higher plants from 74 families. The seed collection includes seeds obtained from the Main Botanical Garden of the Russian Academy of Sciences, collected by Dr. V.L. Tikhonova; orchid seeds; seeds from IPPRAS; and seeds received from Apthecarsky Ogorod at Moscow State University [17]. In addition to freezing new and unique samples, IPPRAS Cryobank aims to monitor the stability of plant material after long-term cryopreservation. For this purpose, seeds of wild, rare, and endangered plants (*C. calceolus* L., *Allium*, and *Veratrum* species) listed in the Red Books of the Russian Federation have been tested for germination after different periods of storage in liquid nitrogen.

## 2. Results

### 2.1. Allium Species

**Figure 1:**
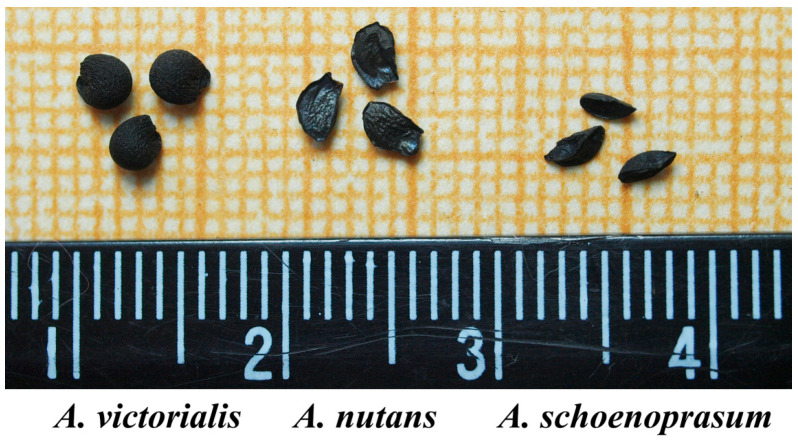
*Allium* species seeds from the collection samples of IPPRAS Cryobank.

We have conducted a metric analysis that showed no significant differences between *A. schoenoprasum* and *A. nutans* seeds. However, seeds of *A. victorialis* were larger and contained the highest amount of free water (WC) (Table 1, Figure 1). The latter seeds also showed the highest germination rates after cryopreservation (Table 2).

**Figure 2:**
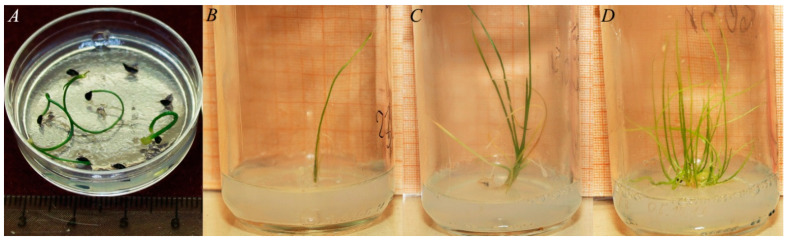
Germination and growing of *A. schoenoprasum* plants after seed cryopreservation. (**A**) Germination of postcryogenic seeds on MS medium [21] containing 0.5 mg/L BAP. (**B**) Plantlets growing on MS medium [21] containing 1 mg/L BAP. (**C**) Plantlets growing on MS medium [21] containing 2 mg/L BAP and 0.5 mg/L IBA. (**D**) Plantlets growing on MS medium [21] containing 4 mg/L BAP, 0.5 mg/L IBA, and 3% sucrose.

**Figure 3:**
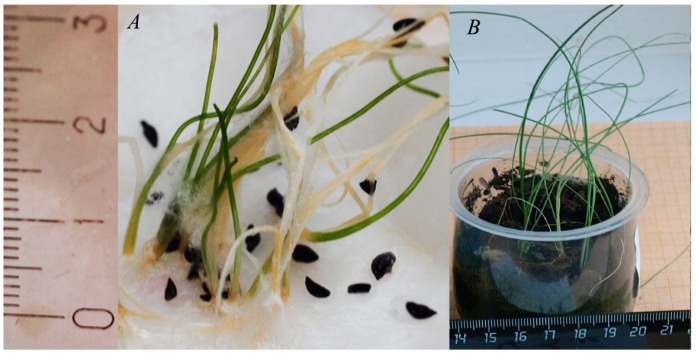
Germination of *A. schoenoprasum* postcryogenic seeds on filter paper and growth of generated plantlets. (**A**) Germinated postcryogenic seeds on wet filter paper. (**B**) Plantlets growing in peat soil in the greenhouse.

**Figure 4:**
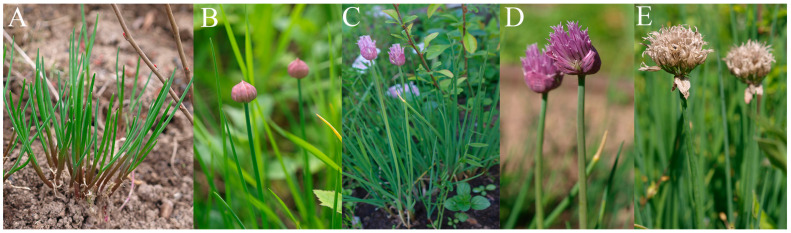
*A. schoenoprasum* plants formed from cryopreserved seeds grown in the open field. (**A**) Young plants transferred to the open field (2021). (**B**) Plants with buds (2023). (**C**) Flowering plants (2023). (**D**) Inflorescences (2023). (**E**) Boxes with seeds (2023).

**Figure 5:**
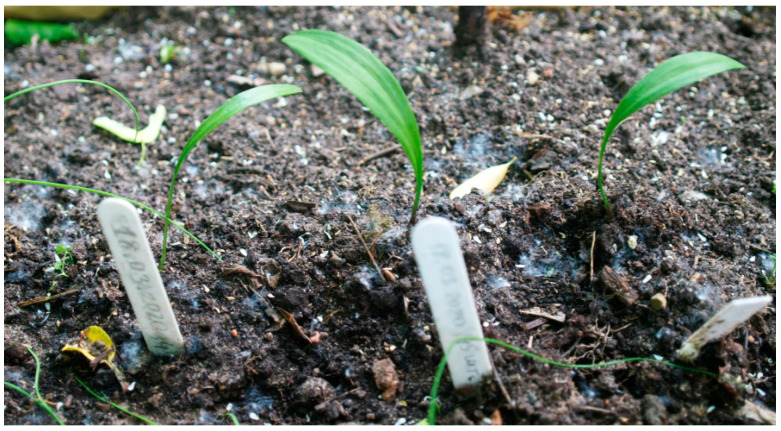
*A. victorialis* plants grown from the seeds after cryopreservation. Plants were transferred to the open field in 2023.

The initial germination rates of *Allium* species were high (72.00–96.55%, Table 2). The germination after cryopreservation decreased, but still remained relatively high. Long-term cryopreservation decreased germination rates as follows: for *A. nutans* from 96.55 to 50.00%, for *A. schoenoprasum* from 72.00 to 62.75%, and for *A. victorialis* from 90.00 to 83.05% (Table 2). *A. victorialis* seeds were germinated on a surface of filter paper. *A. schoenoprasum* seeds were germinated on solid culture medium (Figure 2), as well as on filter paper (Figure 3). *A. nutans* seeds were germinated on culture medium only. In vitro cultivation may increase the possibility of obtaining plants from cryopreserved seeds. However, it is important to select the correct cultivation conditions, which are species-specific. We were unable to obtain mature plants of *A. schoenoprasum* and *A. nutans* from seeds germinated on MS medium [21]. All plantlets died quickly after being transferred to sucrose medium. It is possible that MS medium [21], containing 4 g/L BAP, 0.5 g/L IMC, and 3% sucrose, may not be suitable for this *Allium* species. We observed callus formation on the plantlets of *A. schoenoprasum* and *A. nutans*. This indicated that the hormone ratio in the media was incorrect. Fungal infection was also observed during the germination of *A. schoenoprasum* and *A. victorialis* on filter paper. Despite the infection, we were able to produce mature plants under these conditions. In the field, 17 plants of *A. schoenoprasum* and 3 plants of *A. victorialis* survived. Two *A. schoenoprasum* plants were flowering and fruiting in 2023 (Figure 4). The *A. victorialis* plants have not yet flowered (Figure 5).

### 2.2. Veratrum Species

The initial germination rate of *V. nigrum* seeds prior to cryopreservation was 79.71% (Table 3). Short-term cryopreservation for three days did not lead to a statistically significant change in germination rates (from 79.71 to 82.69%). The initial precryogenic germination rate of *V. lobelianum* seeds was 75.00%. After 19 years of cryopreservation, germination decreased by about 60% and was found to be 14.81% (Table 3).

**Figure 6:**
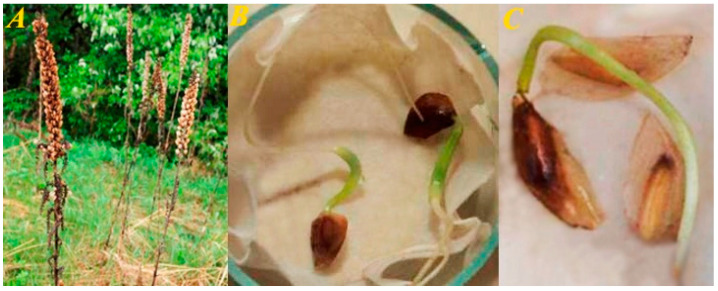
*Veratrum species* cryopreservation. (**A**) *V. nigrum* (boxes with seeds). (**B**) *V. nigrum* plantlets after cryopreservation. (**C**) *V. lobelianum* plantlets after cryopreservation.

We also observed a strong fungal infection, especially in seeds of *V. lobelianum*. Despite this, it was possible to obtain plantlets from cryopreserved seeds (Figure 6). However, we were unable to grow mature plants from the plantlets. The plantlets died after being transferred to the soil.

### 2.3. Stipa Species

**Figure 7:**
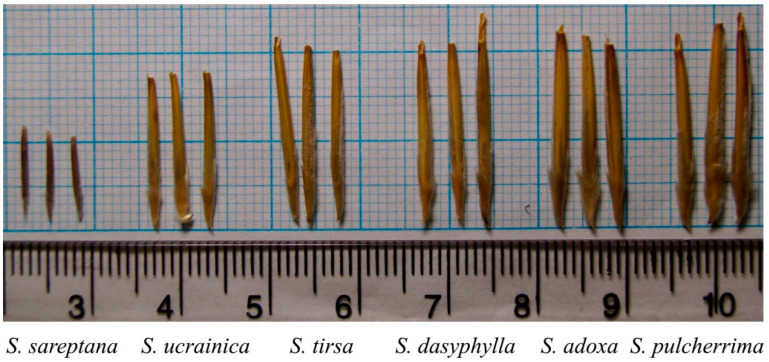
*Stipa*s pecies seeds from the collection samples of IPPRAS Cryobank.

We found that seeds of *S. sareptana* were significantly smaller in length, diameter, and mass; they also showed a lower WC when compared to seeds of other *Stipa* species (Table 4; Figure 7). The WC in seeds of all experimental samples has not exceeded 7%, which is the optimal WC for cryopreservation.

The initial germination rates of *Stipa* species seeds prior to cryopreservation varied from 21.43 to 57.14, while after 1951 days (5 years and 4 months) of cryopreservation, these rates varied in a broader range of 6.98 to 90.00. The highest seed germination levels after cryopreservation were seen for *S. sareptana*; the seed germination rates for the other *Stipa* species were found to be decreased significantly. Treatment with NaOH and H_2_O_2_ stimulators allowed seeds to break out of dormancy and form plantlets. Germination levels of *S. sareptana* and *S. dasyphylla* seeds were increased after H_2_O_2_ treatment, where *S. ucrainica* seeds were able to germinate only after H_2_O_2_ treatment. *S. adoxa* and *S. pulcherrima* seed germination levels were increased after NaOH treatment. At that moment, we ran out of *S. tirsa* seeds and were not able to use them in this set of the experiments (Table 5). Unfortunately, all the plantlets of *Stipa* species obtained during this particular study died after being transferred to the soil.

### 2.4. Cypripedium calceolus L.

**Figure 8:**
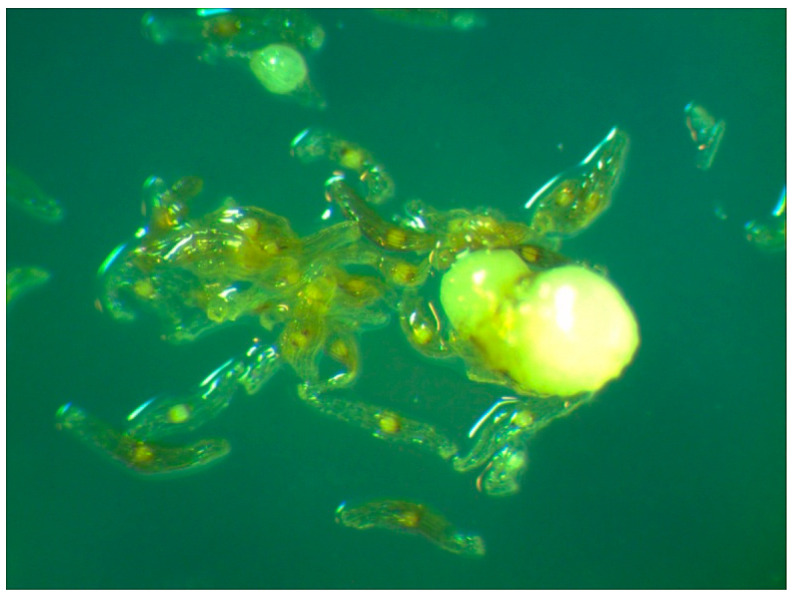
*C. calceolus* protocorm formed on 1/2 MS medium [21].

Petri dishes containing postcryogenic seeds of *C. calceolus* were transferred into the light. Three months later, protocorms were formed. On 1/2 MS medium [21], the highest rate of protocorms was seen (24.98%, Table 6; Figure 8), while on MS medium [21] containing 10% coconut milk, the lowest rate (10.02%) of protocorms was seen (Table 6). On BM1 medium [22], 15.02% of seeds formed protocorms (Table 6). Unfortunately, after 2.5 months on the media, all protocorms turned dark and died. This was most likely due to either poor quality seeds or unsuitable in vitro cultured conditions, or both.

## 3. Discussion

### 3.1. Allium Species

*Allium* species are perennial plants of the *Alliaceae* family that includes vegetables and ornamentals. Modern systematists identified about 900 species of bulbous plants that grow naturally in meadows, steppes, forests, and mountains. Various species of *Allium* contain minerals (potassium, calcium, phosphorus, sodium, and iron), essential oils, saponins, and plant flavonoids, which are substances that have positive effects on human health [23]. Due to these species’ extensive use in agriculture, medicine, and ornamental floriculture, *Allium* collections are included in many genetic banks [24]. Because of the inability of some Allium species to form seeds, the short life spans of seeds (microbiotics), and the impossibility of obtaining a bulbs from seeds in the year of sowing, in vitro technologies using cryopreservation have been developed for the cultivation and conservation of *Allium* species. The best known cryopreserved collections of *Allium* species include the Gene Bank of the National Agrobiodiversity Centre, Suwon, Republic of Korea [25]; the Research Institute of Horticulture, Skierniewice, Poland [26]; and the Leibniz-Institut für Pflanzengenetik und Kulturpflanzenforschung, Regensburg, Germany [27,28]. IPPRAS Cryobank contains seeds of 15 *Allium* species [17]; all these species are included in the regional Red Data Books of the Russian Federation. The germination of three of the *Allium* species, *A. nutans*, *A. schoenoprasum*, and *A. victorialis*, has been investigated in the current study. According to some reports, *A. nutans* and A. *schoenoprasum* have physiological dormancy and require cold stratification [29]. *A. victorialis*, in turn, has morphophysiological dormancy and requires thermal stratification [30]. We decided that stratification could be replaced by the treatment of the seeds with a stimulant, hydrogen peroxide, which is currently used to make them aseptical. The seeds studied here germinated without stratification. It may be assumed that the physiological dormancy of the seeds of the *Allium* species studied is not deep, based on the high rate of germination. According to Levitskaya (2017), seeds with non-deep physiological dormancy are suitable for cryopreservation [31]. We observed this for the studied *Allium* species.

The short-term *cryopreservation* of *A. nutans* and *A. schoenoprasum* seeds was carried out by Golubev (2003) [29]. The seeds were stored for one month at +5 °C, −20 °C, or −196 °C [29]. After cryostorage, it was found that the germination rates of *A. nutans* seeds were 97%, 98%, and 98% and that those of the *A. schoenoprasum* seeds were 72%, 75%, and 70%, respectively. Thus, short-term cryopreservation did not significantly change the germination rates of *A. nutans* and *A. schoenoprasum* seeds [29]. After 30 days of storage in liquid nitrogen vapor, the germination rates of *A. rotundum* seeds increased from 50% to 76%, using slow freezing, and to 80%, using the fast freezing technique [32]. Stanwood and Sowa (1995) [33] tested the germination of *A. cepa* seeds stored at +5 °C, −18 °C, and −196 °C for 10 years. They found that the average germination values of seeds stored at −18 and −196 °C did not decrease, whereas the germination rates of seeds stored at 5 °C decreased from 94% to 68% [33]. The successful cryopreservation of *A. cepa* seeds has also been described by Lakhanpaul (1995) [34]. In our studies, long-term cryopreservation resulted in a decrease in germination (47% for *A. nutans*, 9–12% for *A*. *schoenoprasum*, and only 7% for *A*. *victorialis* (Table 2)). Nevertheless, we consider it a great success to obtain plantlets of *A. nutans* after 17 years of cryopreservation, plants of *A*. *victorialis* after 30 years of cryopreservation, and flowers and seeds of *A. schoenoprasum* after 19 years of cryopreservation (Figure 4 and Figure 5).

### 3.2. Veratrum Species

*Veratrum* species are perennial rhizomatous plants of the family *Melanthiaceae.* This family includes medicinal, poisonous, and insecticidal plants. A total of 25 species (45 according to other sources) were identified that are widespread in Europe, Asia, and North America. *Veratrum* species contain more than 200 alkaloids, including cyclopamine, which was isolated from *V. californicum* and was shown to cause birth defects such as ‘Cyclops’ in livestock [35]. Later, oncologists at Johns Hopkins found that cyclopamine suppressed specific cultured mouse brain tumor cells and human medulloblastoma cells [36]. Subsequently, cyclopamine is currently used as an anticancer drug [37].

*V*. *nigrum* grows on the edges of forests, meadows, and steppe slopes. It is a relic of the xerothermic period, listed in the regional Red Data Books of the Russian Federation. *V*. *nigrum*—based preparations show antihypertensive, anticancer, and antifungal effects. This species contains more than 60 steroidal alkaloids with promising medical uses, which is why it has recently attracted a lot of attention from researchers [38,39]. It is also cultivated as an ornamental plant.

*V*. *lobelianum* grows on dry meadows, floodplain meadows, and glades. It is a relic of the Ice Age and is listed in the regional Red Data Books. *V. lobelianum* is used in Russia as a folk remedy against alcoholism [40]; this remedy often leads to intoxication. In addition, *V. lobelianum* is registered in the Russian Pharmacopoeia and is used in the production of *Veratrum aqua*, which is an alcoholic tincture of rhizomes and roots, equally diluted with water. The main alkaloid constituents of *Veratrum aqua* are gervine, protoveratrin A, and protoveratrin B. According to the literature, gervin has anti-tumor [41,42], anti-inflammatory, analgesic [43], and radioprotective [44] activities. There is a study investigating the cardiotoxic mechanism of gervin, protoveratrin A, and B [45].

According to some literature sources, *V*. *lobelianum* and *V*. *nigrum* require cold stratification [46]. The seeds of *V. nigrum* used in this study were naturally stratified, while the seeds of *V. lobelianum* were not stratified. Just like for *Allium* species, we decided that stratification could be replaced by treating the seeds with hydrogen peroxide. *V. nigrum* belongs to the species with morphophysiological dormancy [47]. We did not find any reports regarding the type of dormancy in *V. lobelianum*. Seed germination of both *Veratrum* species was obtained after cryopreservation, without additional stratification.

The data on the cryopreservation of the seeds of *Veratrum* species are scarce. After 30 days of storage in liquid nitrogen vapor, the germination rate of *V. nigrum* seeds increased from 66% to 76%, using both slow and fast freezing [32]. Levitskaya (2017b) tested the germination rates of *V. nigrum* seeds stored at +5 °C, −20 °C, and −196 °C for 1 month, 3, 6, 9, and 12 years. It was found that seed germination rates decreased from 98.0% to 15.5% when stored at +5 °C, from 98.0% to 93.5% at −20 °C, and from 98.0% to 92.5% during storage at −196 °C for 12 years. When seeds were stored at −196 °C for one month, almost the same germination rate was observed (98.0% vs. 97.3%) [47]. Ma et al. (2006) described the preparation of a cell suspension of *V. californicum*, its cryopreservation, thawing, and rapid recovery [48]. In our work, the short-term cryopreservation of *V. nigrum* did not result in a statistically significant change in germination rates (from 79.71% to 82.69%, Table 3). The long-term cryopreservation of *V. lobelianum* resulted in a 60% reduction in germination from 75.00% to 14.81% (Table 3). Despite the significant decrease in germination values, *V. lobelianum* plantlets were obtained after 18 years of cryopreservation, which is a positive result from our work. *V. nigrum* was found to be tolerant to short-term cryopreservation, thus further cryopreservation experiments of *V. nigrum* are included in our perspective plans.

### 3.3. Cypripedium calceolus

*C. calceolus* is a perennial rhizomatous plant of the family *Orchidaceae.* It is a Euroasian forest species listed in the Red Data Book of the Russian Federation. According to De and Medhi [49] it produces the alkaloid Cypripedin, which is used as an anti-cancer drug in the treatment of lung cancer [50]. *C. calceolus* is the most beautiful northern orchid, cultivated as an ornamental plant. A number of varieties, as well as hybrids, of this plant are available.

*C. calceolus* has been successfully cultivated asymbiotically in vitro. Pauw and Remphrey (1992) studied the germination of three cultivated *Cypripedium* species and found that seed germination rates depended on the year of collection, seed maturity, and culture medium. In most cases, the highest germination levels were observed for immature seeds formed 8 weeks after pollination. For the subsequent development of protocorms, the best medium was Norstog [51], containing one-half macroelements and benzyladenine at 0.2 mg/L [52]. Klavina et al. (2009) planted immature seeds on Norstog medium [51], modified by Pauw and Remphrey (1992), and obtained *C. calceolus* plants in the field three years after the beginning of the experiment [53]. Kozlova et al. (2008) used immature seeds planted on MS medium [21] with 1 g/L of activated carbon and successfully obtained *C. calceolus* plantlets [54]. Jakobson et al. (2009) used immature white-brown seeds planted on Knudson medium [55] and 1/2 MS medium [21] and obtained *C. calceolus* plants in the field three years after the start of the experiment [56]. Znaniecka et al. (2015) used immature seeds to obtain protocorms and further obtained protocorm-like bodies (PLBs) from protocorms that were 1.5 to 2 mm in length. The highest micropropagation rates were found on modified 1/5 MS medium [21] supplemented with 2.0 mg/L TDZ and 2.0 mg/L NAA [57]. Konovalova and Molkanova (2020) used immature seeds (45–60 days after pollination) and concluded that the maximum germination rates were obtained on Harvais medium [58] with supplemented potato, on Chu and Mudge medium [59] supplemented with kinetin, or on Malmgren medium [60] with organic nitrogen, where casein hydrolysate was replaced by sports nutrition micellar casein [61]. Konovalova and Molkanova (2020) also germinated mature seeds of *C*. *calceolus* on Norstog medium [51] with 2.4 mg/L kinetin [61]. The seeds were incubated for 3 months in the dark at +4 °C. When working with mature seeds, the germination level is lower, the time for cold stratification is lost, and the results are unstable, but there is the possibility of the long-term storage of seeds before the experiment, which is particularly important when collecting seeds in field expeditions [61].

In experiments on the asymbiotic germination of *C. calceolus* in vitro, the authors use different media with or without the addition of hormones, but all of them mention that the seeds, after planting on the culture medium, should be placed in the dark for several months. Also, when plantlets are produced in the second year, they need to be placed in the cold at +3 – +5 °C for several months, which simulates winter conditions and is necessary for further root and leaf formation. Many authors recommend adding activated carbon to the medium for plantlets. This is because plantlets produce phenolic exudates in the medium, which need to be absorbed [54,61]. The effect of cold stratification on immature seeds is unclear. Pauw and Kozlova believe that cold stratification is not necessary when using immature seeds [52,54].

We found no data on the successful cryopreservation of *C*. *calceolus* seeds with subsequent thawing and plantlets generation. Previously, the development of the cryopreservation of protocorms and immature *C*. *calceolus* seeds was reported by Znaniecka et al. [57]. We attempted the short-term cryopreservation of *C. calceolus* seeds and produced protocorms after thawing. The highest share of protocorms, 24.98%, was formed on 1/2 MS medium [21] (Table 6). Unfortunately, after 2.5 months, the protocorms died. This was most likely due to poor quality seeds or unsuitable in vitro cultured conditions, or both. We hope that the cause of the death of the protocorm is not related to cryopreservation damage. IPPRAS Cryobank has a positive experience with the cryopreservation of the northern orchids *Dactylorhiza baltica* and *D. maculata* [62], although this does not mean that *C. calceolus* seeds may also be cryotolerant. Further research is required to address this issue.

### 3.4. No Correlation Was Found between the Metric Characteristics of Seeds and Their Cryotolerance

*S. sareptana* seeds are significantly smaller than those of the other *Stipa* species in length, diameter, and weight and, in addition, they have a lower WC (Table 4; Figure 7). *S. sareptana* is the most widespread and drought-tolerant plant of the *Stipa* species studied, classified as a xerophyte [63]. In our previous study, the seeds of *S. sareptana* were found to have the highest germination rates. These rates did not decrease after long-term cryopreservation for 1951 days, in contrast to the other *Stipa* species studied (Table 5) [64]. Based on this, we suggested that the best habitat adaptation and stress tolerance of the particular species may correlate with its high cryotolerance [64]. Although being true for *S. sareptana*, this assumption was not confirmed for the *Allium* species. Among *Allium* species, *A. victorialis* seeds have the highest mass and WC (Table 1). *A. victorialis* prefers shady areas under tree cover and is classified as a mesophyte. Among *Allium* species, *A. victorialis* seeds show the highest germination rates after cryopreservation (Table 2). *A*. *nutans* seeds have smaller sizes and a lower WC than *A.victorialis*. *A. nutans* is the most drought-tolerant of the *Allium* species studied, it grows in meadow steppes, and is classified as a xeromesophyte [63]. After cryopreservation, *A. nutans* seeds germinate at the lowest levels among plants from the *Allium* genum (Table 2). Thus, we did not find any correlation between the metric characteristics of the seeds (size, weight, or WC) and cryotolerance levels. We were also unable to connect the life form of the species, which indicates water requirements, with cryotolerance. Of course, a larger number of species and much larger samples of seeds need to be studied before we may come to this conclusion. However, our data are consistent with the results reported by others. Touchell and Dixon (1993) studied the germination of 68 native Western Australian plant species seeds after storage in liquid nitrogen for two weeks. There were no trends in a species’ ability to survive liquid nitrogen storage and freezing regime, moisture content, seed size, or taxonomic relatedness [15]. Nikishina et al. (2011) compared initial germination and germination after a single freeze in liquid nitrogen of *Triticum aestivum*, *Fragaria vesca*, *Cymbidium mastersii*, and *Dendrobium crumenatum* seeds. They found that *D. crumenatum* seeds, the smallest and driest, have lower germination levels than *C. mastersii* and *F. vesca* seeds [65]. Tikhonova also found that there was no correlation between cryotolerance and the size of the seed, the moisture content, or the location of the seeds’ collection [66].

### 3.5. Orthodox Seed Cryopreservation Problems

There are several factors affecting orthodox seed cryopreservation. Firstly, the inability to predict their tolerance to liquid nitrogen. Studies by other groups [13,15,16,65,66,67,68] conclude that it is not possible, at present, to make predictions regarding the cryopreservation tolerance of a plant species. This ability does not depend directly on the mass, size, or chemical composition of the seeds [15]. Touchell and Dixon (1994) attempted to relate cryopreservation ability to seed lipid content, composition, structure, and amino acid content. They concluded that the successful cryopreservation of seeds is not dependent on a single factor, but rather a combination or interaction of the chemical and physical properties of the seeds. Touchell and Dixon (1994) also suggested that it may be more beneficial in terms of predicting liquid nitrogen tolerance species that their chemical constituents be investigated con-generically, rather than search for trends amongst disparate taxonomic groups [16]. Secondly, finding the optimal moisture content in seeds before cryopreservation. The critical moisture content for seed cryopreservation is species-specific and may even differ within species for different populations [13,66]. Touchell and Dixon (1994) assumed that moisture levels are crucial for cryopreservation; however, the interaction between imbibed water and the other components of a seed during freezing may be more significant for predicting seed survival in liquid nitrogen [16]. The third factor is the initial seed quality and polymorphism. Tikhonova, who was involved in the conservation and reintroduction of medicinal and rare plants for more than 40 years from the 1960s to 2004, noted the issues associated with the polymorphism of wild plant seeds [66]. It is difficult to collect high quality, uniform seeds from the wild. Seeds are contaminated with fungi, vary in weight, size, maturity, dormancy depth, and initial germination. Tikhonova (1992) also noted the different response of seeds from the same collection to hormone treatment and irradiation. Seed polymorphism in nature is necessary for plants, as part of a survival strategy in response to unstable environmental conditions. In addition, seed quality and germination depend on the weather conditions at the time of seed formation [52,66]. Seed quality depends on the biogeocenosis, where the plant was growing, and even on the population of the same species. Poor quality seeds may be damaged primarily at the time of plunging in liquid nitrogen. When it comes to cryopreserving the seeds of wild species, Tikhonova (1992) suggested creating an area that is most suitable for each species (shading, soil composition, and moisture) and then collecting seeds from the grown plants in dry, windless weather [66]. Fourthly, seeds may be desiccation tolerant but sensitive to liquid nitrogen. For example, in the seeds of some *Fabaceae* species, for instance, *Lespedeza cyrtobortrya*, cryopreservation causes the cracking of the seed coat, which can result in up to 79% of abnormal seedlings, with damage to the seedpods, germinal root, and hypocotyls [67]. It is possible that the damage is caused by the high rate of water absorption by the strophiole, resulting in the stretching of the embryo and the formation of cotyledon cracks [69]. Damage is also thought to occur due to differential rates of contraction (compaction) and expansion of embryo tissues during seed cooling and thawing [70]. This form of freeze damage has been noted for seeds of bean, flax, soybean, and radish. The damaged seeds may germinate, but plants cannot be developed from such seedlings [13]. Damage may occur in the first moments of plunging into the liquid nitrogen. Some seeds may be saved from this by slow freezing, pre-treatment with cryoprotectants, or by cutting the seed coat [16]. It is, therefore, recommended that seeds are first screened for short-term cryopreservation via short-term storage in liquid nitrogen (from a few days to a month), then seed germination is checked and compared with the initial germination rates [66]. The fifth factor affecting orthodox seeds’ cryopreservation is their variable depth of dormancy. Dormancy may not be broken by cryopreservation; sometimes cryopreservation may induce secondary dormancy [66,67]. In this case, it is necessary to carry out additional manipulations—stratification, scarification (mechanical or treatment with acids, alkalis, or peroxides), or treatment with stimulating agents (potassium nitrate, hormones, irradiation, etc.) [71]. Finally, seed germination rates may decrease with the duration of cryopreservation. In our study, the highest decrease in germination occurred in *V. lobelianum*, by 60%, from 75.00% to 14.81% (Table 3) and in *A. nutans*, by 47%, from 96.55 to 50.00 (Table 2). Ballesteros and Walters (2011) suggested that the combination of extreme desiccation and extreme cooling may lead to abnormal temperature responses in senescence kinetics, resulting in reduced germination [72]. Levitskaya (2017b) found a correlation between seed dormancy type and germination rates after prolonged cryostorage [47]. Using five species of the genus *Sampanula* as an example, Levitskaya (2015) showed that the deeper the physiological dormancy of the seeds, the faster they senesced at negative (−20 °C) and ultra-low (−196 °C) temperatures. To explain this fact, the author suggested that the development of seeds with physiological dormancy is stopped at an earlier stage, when a sufficient amount or not the whole functional complex of sHSPs (small Heat Shock Proteins) and LEA proteins (Late Embryogenesis Abundant proteins), which are responsible for the resistance of cells to dehydration and dry storage, including low temperature conditions, have not yet been synthesized [73]. In our study, *S. sareptana*, which showed the highest germination rates after cryopreservation, also had the lowest dormancy depth among the *Stipa* species studied. Unfortunately, the dormancy depth *of* the *Allium* and *Veratrum* species was not clearly determined.

One of the solutions to the problem of germination rate loss may be the germination of seeds in vitro and the further cultivation of plants under greenhouse conditions [16,66,67]. In this case, even a few living cells from the embryo may give rise to a whole plant. If the plant is valuable, all the efforts are justified. Each species is unique in nature and no one can assess the true damage of its loss.

## 4. Materials and Methods

### 4.1. Making the Seed and Fruit Aseptic

Seeds of *Allium* and *Veratrum* species were made aseptic by incubating them in 5% hydrogen peroxide solution for 20 min and then washing them three times with sterile distilled water. Immature fruit of *C. calceolus* were made aseptic by incubating them in 96% ethanol for 30 s, then in 3% hydrogen peroxide (H_2_O_2_) solution for 5 min, then washing three times with sterile distilled water.

### 4.2. Seed Germination

Petri dishes with *Allium* and *Veratrum* species and *C. calceolus* seeds were placed under the conditions of a stable temperature regime (20 ± 2 °C) and illumination (2000 lux) at 16 h a day (climatic chamber of the IPPRAS phytotron with automatic air conditioning); Gree, PRC, and fluorescent lighting: LB-40 “OSRAM”, Russia. The seeds were germinated for 30 days. Seed germination rates were determined as the ratio of germinated seeds to the total number of seeds and were expressed as percentages.

### 4.3. Cryopreservation and Thawing of Seeds

Desiccated seeds were placed in cryoampoules (Nunc, Thermo Fisher Scientific, Waltham, MA, USA). The samples in the labeled cryoampoules were then placed in a rack and immersed in liquid nitrogen at −196 °C. Biological cryostorage (XB-0.5; Ural Compressor Plant, Ekaterinburg, Russia) was used for cryopreservation. The seeds were thawed at room temperature. For one experiment, one cryoampoule was thawed without subsequent refreezing.

### 4.4. Morphological Characteristics of the Seeds

To study the morphological characteristics of the seeds, we determined their mass (mg), diameter (maximum value, mm), and length (mm). Measurements were made using a binocular microscope (MBS-10, LZOS, Lytkarino, Russia) and a video camera (Levenhuk C510 NG 5M, Levenhuk, Inc., USA) connected to a laptop computer (Sony VAIO, Azumino, Japan). ScopePhoto 3.1 software (https://scopephoto.software.informer.com/) was used to record the measurements taken. Photographs of plant material were taken using a camera (Sony Alpha SLT-A37, Sony, Tokyo, Japan). Seed weight and water content (percentage of initial weight) were determined using the weight method.

For *Stipa* and *Allium* species, the free water content was determined. For this purpose, samples were weighed on balances (Scout Pro SPU202, OHAUS, Parsippany, USA) before and after drying to a constant weight. Drying was carried out in a drying chamber (80-01, SKTB SPU, Smolensk, Russia) at 104 °C for 24 h. The free water content of the seeds of the *Veratrum* species was not determined, due to the small number of seeds. The seeds of *Veratrum* and *Allium* species were not tested for viability using the TTC test, because their seed coats were dark and no red coloring was visible on them; accordingly, the dormancy was also not determined. The morphological characteristics of *Veratrum* species seeds were not analyzed, due to their small number. The morphological characteristics of *C. calceolus* seeds were not analyzed, due to their microscopic size and preciousness.

### 4.5. Statistics

The tables present mean values with their standard deviations, or percentage values with their standard errors of the proportion. Statistical analysis was performed using an ANOVA test. The experimental data demonstrated a significant difference at *p* < 0.05.

### 4.6. Allium Species

In our work, we have used the orthodox seeds of three rare *Allium* species, *A. nutans* L., *A. schoenoprasum* L., and *A. victorialis* L. The place of collection and storage of seeds of *Allium* species is shown in Table 7. In each experiment, about 50 seeds of each species were used.

To test germination after cryopreservation, aseptic seeds of *A. schoenoprasum* and *A. nutans* were placed on MS medium [21] containing 0.5 mg/L BAP, in Petri dishes. The dishes were then placed in the climatic chamber of IPPRAS. After two weeks, the seeds of both species germinated and were then transferred to MS medium [21] containing 1 mg/L BAP. After a period of three months, the plantlets were transferred to MS medium [21] containing 2 mg/L BAP and 0.5 mg/L IBA. Following an additional three months, the plantlets were then transferred to MS medium [21] containing 4 mg/L BAP, 0.5 mg/L IBA, and 3% sucrose.

To test germination after cryopreservation, aseptic seeds of *A. schoenoprasum* and *A. victorialis* were placed in Petri dishes on wet filter paper, under sterile conditions. Petri dishes were placed in the climatic chamber of IPPRAS. After one month, germinated seeds were planted into pots with peat soil mixture, covered with plastic cups to achieve high humidity, and were transferred to the greenhouse of IPPRAS. Two months later, the plantlets were planted in the open soil.

### 4.7. Veratrum Species

In our work, we have used the orthodox seeds of two rare medicinal *Veratrum* species—*V. lobelianum* Bernh. and *V. nigrum* L. The seeds of *V. lobelianum* were collected by V.L. Tikhonova in 1998 on the Tsitsin Main Botanical Garden RAS. Before cryopreservation, the seeds were stored for two years in a refrigerator at +4 °C in the dark and at a relative humidity of 40–60%. The seeds of *V. nigrum* were collected in May 2018 by M.V. Sementsova in the Moscow region, in the valley of the Oka River in the Prioksko-Terrasny Reserve, and amounted to 123 pieces [74]. The seeds underwent natural stratification during the winter of 2017/2018. The seeds of *V. nigrum* were stored in the dark, at room temperature (20–25 °C) and 40–60% humidity for three months, before cryopreservation. For the experiment, 27 seeds of *V. lobelianum* and 52 seeds of *V. nigrum* were thawed. To test initial germination before cryopreservation, 52 seeds of *V. lobelianum* and 69 seeds of *V. nigrum* were used. Two seeds of *V. nigrum* were damaged and did not participate in the experiment [74].

To test germination before and after cryopreservation, we germinated aseptic seeds of *V. nigrum* and *V. lobelianum* on filter paper moistened with sterile thawed water, in Petri dishes. Petri dishes were placed in the climatic chamber of IPPRAS. After one month, germinated seeds were planted into pots with peat soil mixture, covered with plastic cups for high humidity, and were transferred to the greenhouse of IPPRAS.

### 4.8. Stipa Species

In our work, we have used the orthodox seeds of six rare *Stipa* species—*S. sareptana*, *S. ucrainica*, *S. tirsa*, *S. dasyphylla*, *S. adoxa*, and *S. pulcherrima*. The seeds were collected by M.I. Antipin in July–August 2015, in the Botanical Garden of the Southern Federal University in Rostov-on-Don, Russia. They were stored in the dark, at room temperature, and frozen in February 2015. Approximately 50 seeds of each species were used in every experiment. *Stipa* species characteristics, collection site, seed sterilization, seed viability, germination test, and treatment with different stimulators were previously reported [64].

### 4.9. Cypripedium calceolus L.

In our work, we used the orthodox seeds of endangered, ornamental orchid *C. calceolus*. An immature fruit was collected by G.L. Kolomeitseva in September 2022 in the Yaroslavl region, Russia. Before cryopreservation, the fruit was stored for three months in a refrigerator at +4 °C, in the dark, with a relative humidity of 40–60%. After the fruit was made aseptic, light-brown seeds were extracted under sterile conditions. For cryoconservation, the seeds were partially dehydrated in a laminar airflow chamber at room temperature and 40–60% relative humidity for 4 h [75]. The dehydrated seeds were placed in a cryoampule and were frozen in liquid nitrogen for three days. The number of seeds was determined to be several thousand. The germination of *C. calceolus* seeds before cryopreservation could not be determined, as there was only one fruit. After cryopreservation, the seeds were equally distributed into three parts and were then placed on Petri dishes containing one of the following three media: 1/2 MS, MS supplemented with coconut milk 10% [21], or BM1 [22]. The pH of the culture media was measured before autoclaving and was determined to be between 5.8 and 6.0. The media were autoclaved at 121 °C for 15 min. Petri dishes containing seeds were placed in a dark room at 22–25 °C for three months. Then, Petri dishes were moved to the climatic chamber of IPPRAS.

## 5. Conclusions

As a result of the studies carried out, it was possible to demonstrate the efficiency of the cryopreservation of three *Allium* species—*A. nutans*, *A. schoenoprasum*, and *A. victorialis*—and two *Veratrum* species—*V. nigrum* and *V. lobelianum*. Plantlets were obtained from all the above species. *A. schoenoprasum* and *A. victorialis* were able to form plants from plantlets. Plants of *A. schoenoprasum* flowered and fruited. Protocorms of *C. calceolus* were produced after 3 days of cryopreservation, but, unfortunately, died quickly. This was most likely due to either the poor quality of seeds or unsuitable in vitro cultured conditions, or both. Methods for the culturing and cryopreservation of *C. calceolus* need to be further improved.

## Figures and Tables

**Table 1:** Metric characteristics of *Allium* species seeds.

Species	Length, mm	Diameter, mm	Average Weight of a Seed, mg	Free Water Content (WC), %
*A. nutans*	3.85 ± 0.15	2.11 ± 0.19	2.61 ± 0.14	7.11 ± 0.48
*A.* *schoenoprasum*	3.05 ± 0.17	1.66 ± 0.20	1.25 ± 0.11	5.24 ± 0.24
*A.* *victorialis*	3.57 ± 0.12	3.49 ± 0.13	7.82 ± 0.37	10.57 ± 0.53

**Table 2:** *Allium* species—duration of cryopreservation, germination rates before and after cryopreservation, and number of plants yielded.

Species	Cryo Date	Thawing Date	Cryo Duration	Germination before Cryo, %	Germination after Cryo, %	Germination Conditions	Mature Plants
*A. nutans*	20 July 2000	5 April 2018	17 years 8 months 16 days	96.55 ± 2.40 ^a^	50.00 ± 6.93 ^e^	medium	-
*A.* *schoenoprasum*	20 July 2000	5 April 2018	17 years 8 months 16 days	72.00 ± 6.35 ^cd^	62.75 ± 6.77 ^de^	medium	-
		11 February 2020	19 years 6 months 22 days	72.00 ± 6.35 ^cd^	60.00 ± 6.93 ^de^	filter paper	17
*A.* *victorialis*	5 June 1989	18 March 2020	30 years 9 months 13 days	90.00 ± 4.24 ^ab^	83.05 ± 4.89 ^bc^	filter paper	3

Different lowercase letters in the table indicate about significant differences at *p* < 0.05 for experimental datas.

**Table 3:** *Veratrum* species—duration of cryopreservation, germination rates before and after cryopreservation, and number of plants yielded.

Species	Cryo Date	Thawing Date	Cryo Duration	Germination before Cryo, %	Germination after Cryo, %	Germination Conditions	Mature Plants
*V. nigrum*	20 July 2018	23 July 2018	3 days	79.71 ± 4.84 ^a^	82.69 ± 5.25 ^a^	filter paper	-
*V. lobelianum*	20 July 2000	23 July 2018	18 years 3 days	75.00 ± 6.00 ^a^	14.81 ± 6.87 ^b^	filter paper	-

Different lowercase letters in the table indicate about significant differences at *p* < 0.05 for experimental datas.

**Table 4:** Metric characteristics of *Stipa* species seeds.

Species	Length, mm	Diameter, mm	Average Weight of a Seed, mg	Free Water Content (WC), %
*S. sareptana*	11.05 ± 0.36	1.15 ± 0.16	3.55 ± 0.11	5.13 ± 0.51
*S. ucrainica*	17.45 ± 0.41	1.70 ± 0.21	16.00 ± 0.87	6.62 ± 0.49
*S. tirsa*	20.85 ± 0.54	1.80 ± 0.18	22.40 ± 1.46	6.77 ± 0.87
*S. dasyphylla*	21.10 ± 0.63	2.10 ± 0.09	23.85 ± 2.03	6.94 ± 0.94
*S. adoxa*	22.15 ± 0.71	2.20 ± 0.11	30.65 ± 3.18	5.55 ± 0.62
*S. pulcherrima*	22.75 ± 0.75	2.30 ± 0.11	25.40 ± 1.85	6.34 ± 0.78

**Table 5:** Germination of *Stipa* species seeds before and after cryopreservation.

Species	Germination Rates before Cryopreservation, %	Germination Rates after Cryopreservation, %
	Room T (20–25 °C) (6 Months) + Stratification 42 Days	Cryopreservation in Liquid N_2_ (1951 Days) + Pre-Treatment with NaOH or H_2_O_2_
*S*. *sareptana*	57.14 ± 6.61 ^b^	90.00 ± 4.24 ^a^ (H_2_O_2_)
*S*. *ucrainica*	43.14 ± 6.94 ^bc^	7.02 ± 3.38 ^f^ (H_2_O_2_)
*S*. *tirsa*	32.00 ± 6.60 ^cd^	-
*S*. *dasyphylla*	28.30 ± 6.19 ^cd^	11.32 ± 4.35 ^ef^ (H_2_O_2_)
*S*. *adoxa*	21.43 ± 5.48 ^de^	12.96 ± 4.57 ^ef^ (NaOH)
*S*. *pulcherrima*	56.14 ± 6.57 ^b^	6.98 ± 3.88 ^f^ (NaOH)

Different lowercase letters in the table indicate about significant differences at *p* < 0.05 for experimental datas.

**Table 6:** *C. calceolus*—duration of cryopreservation and formation of protocorms.

Species	Cryo Date	Thawing Date	Cryo Duration	Culture Medium	Protocorm Formation Rate, %
*C. calceolus*	5 December 2022	8 December 2022	3 days	1/2 MS	24.98 ± 1.35 ^a^
				MS + coconut milk	10.02 ± 0.89 ^c^
				BM1	15.02 ± 1.11 ^b^

Different lowercase letters in the table indicate about significant differences at *p* < 0.05 for experimental datas.

**Table 7:** *Allium* species seeds characteristics—collection site, collection date, storage conditions and duration of *Allium* species before cryopreservation, and number of seeds used.

Species	Collection Site	Collection Date	Storage Conditions and Duration	Number of Seeds in the Germination Test	
				before Cryo	after Cryo
*A. nutans*	BG RAS (collected by Tihonova V.L.)	1998	refrigerator +4 °C, darkness, 2 years	58	52 medium
*A.* *schoenoprasum*	BG RAS (collected by V.L. Tihonova)	1998	refrigerator +4 °C, darkness, 2 years	50	51 medium
					50 filter paper
*A.* *victorialis*	Moscow Region (collected by T.V. Daletskaya)	1987	20–25 °C, 40–60% humidity, darkness, 2 years	50	59 filter paper

BG RAS-The Tsitsin Main Botanical Garden of the Russian Academy of Sciences.

## Data Availability

The data presented in this study are available upon request from the corresponding author.

## Abbreviations

BAP—6-benzylaminopurine; IBA—Indole-3-butyric acid; TDZ—Thidiazuron; NAA—1-Naphthylacetic acid; MS medium—Murashige and Skoog medium; BM1 medium—Basic Medium; WC—free water content; IPPRAS—Institute of Plant Physiology, Russian Academy of Sciences.
